# A qualitative study to explore strategies to improve the Road to Health Application for maternal and child health outcomes in South Africa

**DOI:** 10.3389/fdgth.2022.1094754

**Published:** 2023-03-09

**Authors:** Janan Janine Dietrich, Lerato Tsotetsi, Thenjiwe Dubazane, Gugulethu Tshabalala, Boitumelo Maimela, Martin Weiss, Mamakiri Mulaudzi

**Affiliations:** ^1^ Health Systems Research Unit, South African Medical Research Council (SAMRC), Bellville, South Africa; ^2^ Perinatal HIV Research Unit (PHRU), School of Clinical Medicine, Faculty of Health Sciences, University of the Witwatersrand, Johannesburg, South Africa; ^3^ African Social Sciences Unit of Research and Evaluation (ASSURE), Wits Health Consortium, Faculty of Health Sciences, University of the Witwatersrand, Johannesburg, South Africa; ^4^ Jembi Health Systems, Cape Town, South Africa and Jembi SAMRC Collaborating Centre in Digital Health Innovation, Cape Town, South Africa

**Keywords:** Road to Health Application, Road to Health App, South Africa, mobile health, child immunization record, maternal and child health, Digital Health, e-health

## Abstract

**Background:**

The Road to Health Application (RTHA) is essentially a digitalized version of the National Department of Health’s Road to Health book, and a hand-printed booklet given to mothers at the birth of each baby. The RTHA, like the booklet, provides guidelines for maternal and child health outcomes, with the goal of creating a database of children and caregivers in South Africa and teaching them how to raise a healthy child. This paper explored potential barriers and enablers to using the RTHA in the South African context based on user experiences.

**Methods:**

Using a qualitative design, we conducted 50 serial interviews (two separate interviews, 1 month apart). Through convenience, sampling eligible participants were 18 years  or older women who were pregnant and/or had a child under the age of 5 years. Participants included 25 existing users and 25 new users of the RTHA, who owned android smart phones at enrollment. Existing users were recruited telephonically through the National Department of Health database, and new users were approached at the antenatal care unit and wellness baby clinic (women with children under 5 years) at the Chris Hani Baragwaneth Academic Hospital. Upon enrollment, participants completed a brief survey on sociodemographics and mobile phone use, and thereafter, they had a baseline interview followed by a telephonic interview 1 month later. A semistructured interview guide was used to explore barriers, enablers, and the usability of the RTHA. Using thematic data analysis, we identified enablers and barriers to the use of the RTHA.

**Results:**

A third (33%) of all participants reported IsiZulu as their main language of communication, and 6% of the participants reported English as their main language of communication. The RTHA was an important addition to the booklet that helped keep new mothers informed about child immunization and provided important information about healthy child rearing practices. However, multiple barriers were cited to using the RTHA; these included the fact that the app was only available in two languages, high data costs, lack of access to smart phones, and app functionalities. The enablers to using the RTHA included the accessibility of important information regarding prenatal and postnatal childcare.

**Conclusion:**

This study gives insight into the barriers and enablers from the end-user perspective to improve the RTHA for future use in South Africa and offers guidance on how to improve the RTHA to be more user-friendly, which could increase its usability among mothers. It further emphasizes the need to consider the challenges experienced by users in South Africa when developing future mobile health interventions to increase uptake.

## Introduction

More than 1 million children are born in South Africa every year (1). Ideally, all children should grow up with equal opportunities for health and physical success, from early childhood development services like supportive nutrition and healthcare to support for primary caregivers and social services to equitable educational opportunities. A lack of such equity in achieving a healthy life, unhindered by the negative consequences of social determinants of health can lead to detrimental economic, physical, educational, and mental outcomes, and by addressing health equity, the playing field is more level in allowing children from all background success, happiness, and health (2).

In line with the National Integrated Early Childhood Development Policy of 2015, (3) the South African Department of Health (NDoH) pursued policy to provide comprehensive, responsive caregiving and health support and sought to shift the focus of health initiatives from survival to facilitating opportunities for children to thrive. The Under 5 Side by Side Campaign, a bold and ambitious intervention targeted at unifying health, nutrition, and development of children younger than 5 years (4), helps address this and encourage more parity among the million children born in South Africa every year. The Under 5 Side by Side Campaign “focuses on the supportive relationship between child and caregiver, as well as the relationship with practitioners, including health-care workers, who help and advise the caregiver” (4) and utilizes/implements the Road to Health booklet in its health targets to empower parents and caregivers with childcare information and help track immunization among children under five. The NDoH envisioned that with this intervention in place, the benefit to each individual child related to childhood services will have positive long-term and short-term effects and will result in a more prosperous and equal society.

The Road to Health booklet records immunizations and vaccinations as a health record and is given to mothers at birth to track weight, height, and immunizations to ensure safety from disease and measure healthy growth. Building off the successes of their MomConnect (5) programme, the NDoH launched an application (app) version or e-version of the Road to Health Booklet in the online Play-store in February 2019. The Road to Health App (RTHA) now exists as a digital application compatible with android-enabled smartphones (6) and allows for interactions between healthcare workers, healthcare services, and caregivers with the overall intent of allowing a parent and/or caregiver to view childcare information and update their child’s health record more easily. This digitization of the progress made by the physical Road to Health booklet mitigates the risk of loss of paper booklets and may help address some challenges to immunization in the African region, including organization challenges (logistics, sustainable funding, vaccine stock-outs, data issues, laboratory infrastructure (7), all while expanding and facilitating communication between caregivers and knowledgeable health professionals and empowering caregivers with accurate, practical childcare information. Other barriers to immunization include “lack of knowledge of immunization, distance to access points, financial deprivation, lack of partner support, and distrust in vaccines and immunization programs,” and challenges including “number of offsprings, lifestyle, migration, occupation, and parent’s forgetfulness, inconvenient time, and a language barrier” (8). By opening the line of communication to healthcare professionals through digital means, the RTHA addresses challenges perpetuated by a lack of knowledge and reliable information, and can be expanded to help address the physical or tangible barriers cited, such as distance to an access point, by directing caregivers to appropriate resources.

Although the RTHA is available for use, information on user feedback is limited. To ensure a user-engaged approach, we engaged existing and potentially future users of the RTHA by way of serial interviews aimed at identifying potential areas for growth and improvement of the application. By assessing the application functionality, practicality, and ease of use. The application can be modified to better engage users by increasing functionality, utility, and app flow and more effectively fulfilling the goals of the RTHA. Therefore, the aim of this study is to determine the barriers, enablers, and overall usability of the app for future uptake.

## Methods

### Research design

We used a qualitative approach by conducting 50 serial interviews with pregnant women and mothers who had children younger than 5 years. Serial interviews occur when the same participant is interviewed more than once (Read, 2018; Murray et al., 2009). We use this method to achieve our study objectives by asking new and existing users of the newly launched RTHA to provide feedback on their experiences with downloading and using the RTHA at study enrollment and after a month period.

### Participant sampling

A sampling strategy combining convenience and purposive sampling was used to enroll 50 women who were pregnant and/or had a child under the age of 5. Participants included 25 existing users and 25 new users of the RTHA. Eligible participants were adult women ages 18 and older, pregnant or having children under the age of 5, and owning an Android smartphone. Men and caregivers were excluded.

### Setting

The Perinatal HIV Research Unit (PHRU), situated in Soweto, an urban African township, 15 km to the southwest of Johannesburg, conducted the study. The population of Soweto is estimated at the census to be 1.5 million; (9) although the study was conducted by the PHRU, participants were recruited through the National Department of Health infrastructure and through active recruitment at the Chris Hani Baragwanath Academic Hospital in Soweto, South Africa.

### Study procedures

Interviews were performed by trained female social science research assistants. Two interviews with each participant were conducted in English and the local languages most spoken in Soweto, i.e., isiZulu and Sesotho. Existing users participated in two telephonic interviews; the first interview was conducted at enrollment, and the follow-up interview was done 1 month after the enrollment date. Both telephonic and in-person interviews were audio-recorded for later transcription to inform data analysis. All participants were reimbursed with ZAR50 (∼3.55 USD at time of writing) airtime for each interview.

New user participants were approached at the antenatal care unit for pregnant women and the well-baby clinic for participants with children under 5 years at the Chris Hani Baragwaneth Academic Hospital. Upon agreement, participants were directed to a private space in the clinic and provided with detailed information about the study to be able to provide signed, informed consent. After obtaining consent, participants were connected to the study *via* wireless fidelity (Wi-Fi) to download the RTHA. Participants were guided through the RTHA registration process, creating profiles and showing the content within the application. Thereafter, participants were requested to complete a brief survey on sociodemographics, mobile phone ownership, and use. Following the surveys, in-depth interviews were conducted to obtain feedback about the RTHA download and registration process, navigation, and the content of the application.

For recruitment of existing users, the investigator obtained a list of contact numbers of existing registered users of the RTHA from the South African NDoH. Using the list of telephone numbers, a standardized text message approved by the Ethics Committee was sent sequentially to all users to determine their interest in participating in the study. Users were able to send a response to a dedicated study phone to express their interest. Study staff contacted those who showed interest telephonically to provide more information about the study. Participants who chose to continue with study participation were asked to provide informed consent verbally for both their participation and the audio recording of the interview. Participants’ verbal consent was audio-recorded as a formal record of informed consent. Participants were assigned a study identification number; however, participant’s name and surname were stated on the verbal consent. Participants were finally asked to complete a brief questionnaire on sociodemographics and mobile phone ownership and use, and the in-depth interview was administered with a follow-up telephonic interview a month after the baseline interview.

### Measures

All participants were asked to respond to a brief survey to assess sociodemographics and answer questions regarding language, level of education, mobile phone use, and data access. A semistructured interview guide was used to explore barriers, enablers, and the usability of the RTHA. Furthermore, the interview guide probed participants about their experiences with downloading and using the RTHA.

### Ethical considerations

Study procedures were approved by the University of Witwatersrand Human Research Ethics Committee, the Medical Advisory Committee of the Chris Hani Baragwaneth Academic Hospital, and the South African National Department of Health. Existing user participants provided verbal informed consent that was audio-recorded and new users provided written informed consent. Participants received R50 (∼3.55 USD at time of writing) of airtime as compensation for their time and participation.

### Data analysis

Survey data were entered into an Excel spreadsheet and analyzed using Microsoft Excel. Descriptive data were summarized using counts and percentages. The in-depth interviews were audio-recorded and transcribed verbatim. The overall data analysis was conducted using Framework Analysis (10). A codebook was created through incorporating inductive and deductive approaches. The senior researcher reviewed the codebook after this process to allow any dissimilarities to be resolved. The codebook and transcripts were uploaded to NVivo, a qualitative data analysis software produced by QSR International (11). Thereafter all of the transcripts were coded by two trained researchers by focusing on barriers, enablers, and the usability of the RTHA.

## Results

### Participant characteristics

A third (33%) of all participants reported IsiZulu as their main language of communication, while 6% reported English as the main language of communication. Most participants (92%) completed grade 12, and of the 46 that completed grade 12, 34 (73.9%) went on to attend post-high school training, and 4 (10%) of the participants did not complete high school (Table 1).

**Table 1:** Sociodemographics.

	*n* = 50	New users, *n* = 25	Existing users, *n* = 25
Level of education*			
Incomplete high school	4 (8%)	3 (12%)	1 (4%)
Complete high school	27 (54%)	15 (60%)	12 (48%)
Post-high school training	18 (36%)	6 (24%)	12 (48%)
Language			
IsiZulu	16 (32%)	9 (36%)	7 (28%)
Setswana	9 (18%)	3 (12%)	6 (24%)
Sesotho	7 (14%)	3 (12%)	4 (16%)
SiSwati	4 (18%)	2 (8%)	2 (8%)
Xhosa	3 (6%)	1 (4%)	2 (8%)
Xitsonga	3 (6%)	2 (8%)	1 (4%)
Sepedi	3 (6%)	1 (4%)	2 (8%)
English	3 (6%)	2 (8%)	1 (4%)
Venda	2 (4%)	2 (8%)	—

* One new user participant did not answer question: level of education.

### Barriers to app usage

#### Mobile phone use and data access

Survey data showed that 40% (20) of the participants bought their own mobile data, with 32% (16) of the participants having access to public/work Wi-Fi and only 18% having access to home Wi-Fi (Table 2). The majority of the participants reported spending 5–7 h on average a day on their mobile phones, with 34% reporting spending more than 8 h. The rest of the participants (24%) reported spending on average 2–4 h on their mobile phones, and only 6% reported spending 0–1 h on their mobile phones. Participants reported using mobile phones to send and receive messages, make and receive phone calls, access the internet, and use social media (Facebook, Twitter, Instagram, WhatsApp, and WeChat). Mobile phones were also reported to be used for entertainment purposes (YouTube, listening to music, playing games), banking purposes, and accessing emails.

**Table 2:** Mobile phone and data access.

	*n* = 50	New users total, *n* = 25	Existing users total, *n* = 25
Own phone	50 (100%)	25 (100%)	25 (100%)
Android phone	49 (98%)	25 (100%)	24 (96%)
Change numbers frequently	8 (16%)	3 (12%)	5 (20%)
Who pays for data?			
Themselves	20 (40%)	11 (44%)	9 (36%)
Parent	6 (12%)	3 (12%)	3 (12%)
Partner	14 (28%)	6 (24%)	8 (32%)
Combination of all three	10 (18%)	5 (20%)	5 (20%)
Access to Wi-Fi			
Home Wi-Fi	9 (18%)	5 (20%)	4 (16%)
Work/public Wi-Fi	7 (14%)	3 (12%)	4 (16%)

For the interview data, the majority of both new and existing users mentioned lack of access to smartphones as a potential barrier that would discourage other mothers from downloading and using the RTHA. Participants reported that although having the information readily available on their devices through the RTHA would be beneficial to the community; they had concern that most mothers in the community do not have access to smartphones, which would affect their ability to use the RTHA. Other participants further noted that some mothers in the community who do have smartphones are likely to not have devices that meet the minimum requirements for downloading the app, i.e., their phones could have Android versions lower than 4.1 and/or an iOS or using other software not yet supported by the application.

 “I think for others it’s just a matter of phone [for instance not a smartphone] that they are using it’s probably not compatible to actually download the App, secondly it could be that they don’t understand exactly how you can download the App or why you need it if you have the book [Road to Health Booklet]”—RTHA-13 (existing user)

Most participants stated that they often go for long periods without mobile data, which consequently made it redundant for them to have the app on their phone if they were unable to use it. Participants therefore reported that the financial implications associated with mobile data would discourage potential users from engaging with the RTHA as they might not have the capacity to keep up with the data costs associated with regular use.

“Well some of them maybe we don’t know how to use smartphones or who might have problems with regards to network where they are staying or they don’t have money for data, that’s…I believe that could be one of the reasons but if somebody has got the resources it won’t be a problem in terms of data, network, smart phone, they won’t have a problem”—RTHA-03 (existing user)

### App usability

Participants reported that a few attributes of the RTHA affected the engagement and navigation of the users, some of these involved technical difficulties, a lack of backup of important information, and the general navigation panel that discouraged participants from using the RTHA more frequently.

#### Technical difficulties

Existing users cited multiple technical difficulties that they experienced when using the RTHA that acted as barriers to regular use. One of the major issues reported was that the RTHA would occasionally take a long time to open and would display error messages such as “fetching the caregiver” and some participants noted that sometimes the app would have difficulties continuing to the next page. For new users, the app sometimes did not allow them to create new caregiver/child profiles but would only show content that did not involve personalized features.

“That’s where you get stuck to move on to the next stage because I went in to log in and I put in the correct credentials, I even said unhide my password so that I can see if I have spelt it wrong but still same message [appeared that says] invalid credentials so that’s when it stops I can’t go to the next level [page]**—**RTHA-02 (existing user)

#### Lack of backup

Eight (16%) of the participants reported changing numbers regularly, and new users reported that the RTHA currently has no functionality that allows one to store backup information. Other existing user participants noted that when they changed SIM cards or phones, the app required them to reenter all their information and that it would not save the child’s weight. Participants further reported that this acts as a barrier because by the time they need to reenter the information, they might have forgotten some of the important details required, and additionally reentering the data was perceived to be time-consuming. Participants further suggested that having their information backed up would be beneficial to the NDoH, as they could potentially use this feature as a backup system for the database for child immunization profiles.

“I: So, you think that the App should be able to store… your information so that even if you change your phone you can continue with the information you had on the App?

R: I mean now I need to start afresh and put that she [the baby] last went for immunisation in January and got this type of injection … whereas if it will have a backup to say you last got this kind of immunisation in June”—RTHA-17 (existing user)

“Sometimes I will try to save the baby’s weight then it will not save”—RTHA-01 (existing user)

#### App design and navigation

Existing users were further dissuaded by some of the esthetics of the app, noting that it had too much text and offered little graphics. Additionally, participants reported that having graphics would be helpful for mothers who have a low literacy level, as it would enable them to make sense of the information offered, even though they cannot read at the level presented. The RTHA navigation panel was perceived as complicated by the participants, particularly by first-time users and those who did not complete high school. Therefore, participants suggested that the navigation menu and some of the wording on the app could be revised to make them easier to understand. Participants reported that updating children’s profiles can be complicated if you do not have the information and/or tools required to measure anthropometric indicators such as height, weight, and the mid-upper arm circumference as required on the app.

 “I was also confused when it said household and then I see where it says I need to add my name and add the child’s name, you know I thought it was talking about somebody else when it said household”—RTHA 03 (existing user)

“I found that it’s not straightforward to use the app. You have to play around to figure out what is happening and how to open, different menus and options”—RTHA 23 (existing user)

### Language

Currently the RTHA is available only in isiZulu and English, and this was perceived as a barrier given that South Africa has 11 official languages. Participants reported that inclusivity was limited, and that the absence of other languages would discourage potential users who do not understand English and isiZulu from engaging with the app. Moreover, potential users in rural areas who do not go to the clinic often would have difficulties using the app without assistance. Most of the participants suggested that the app should have all the official languages of South Africa to ensure that mothers and pregnant women who are not fluent in English and/or isiZulu can be accommodated. It was reported that this could offer an opportunity for people to have the information presented in their own language to increase comprehension and reduce the possibility of other language groups feeling neglected.

“It does not sit well with me, because there is no Tshivenda or Tsonga but in everything, we find that English and Zulu languages are in the forefront. Please also consider us, that there are others from Venda or even here in Joburg who do not know how to read English though they might be able to speak it. So how will they manage, and you could find that while you are busy downloading the app once they realise that it is only in English and Zulu, they might just refuse to continue with the process and say that they are fine. Then you would think that she is being rude, but you [are] also excluding us”**—**RTHA-06 (new user)

 “You know sometimes English can have certain words that you don’t really know what they mean, and you can miss important information so if government can … it would be a good idea to actually include all languages”—RTHA-13-(existing user)

#### No content updates

The RTHA offers standardized information regarding the development of the baby, nutrition, and immunization. However, this was observed to be a barrier mostly for existing users who had more time to engage with the app. Existing users reported that the information provided was not updated per profile or the age of the baby. Therefore, participants had concerns that once they have read the information presented on the app; there will be no need to continue logging o except to update the vaccination tracker. Participants further stated that the information on the app comes across as repetitive and is not personalized according to individual profiles. Other participants stated that they would eventually uninstall the app after reading through all the information.

“I think there would be maybe easily don't update information. If you find that every time we go into the app, you find out repetitive information so I think they must update that information so that it keeps you yearning to go back and learn more”—RTHA-22 (existing user)

“I've found that there's not many like updates that come through so even the news feed is not like their new information that's updated regularly. I only use the app if there is something I want to add like the vaccination, because once you've read the danger signs, it's not like the they are gonna change if you keep going to look or so it’s only when I need to update information”—RTHA-23 (existing user)

### Features and functions of the app

Some of the participants using the RTHA noted that not having reminders or notifications from the app reduced their engagement, as most mothers mentioned that having reminders about new information or vaccination appointments would encourage them to use the app often. Both new users and existing users further stated that being able to use the RTHA for clinic appointments would be convenient for them as they sometimes forget the Road to Health booklet at home but always have their phones with them. Therefore, this was seen as a gap and a point of improvement for the NDoH to scale up and incorporate the app feature into the regular clinic visits.

“I find it absolutely useless to have the app on my phone that every time I go to the clinic, I still have to carry the book**”—**RTHA-29 (existing user)

“I want to know more about my baby. When I encounter a problem, I will be able to read up more on it and what I can do when the baby is sick”—RTHA-22 (new user)

### Postnatal and antenatal knowledge

Participants noted that they would like the RTHA to be implemented in a manner that would provide emotional and physical support for mothers as a strategy to improve postnatal care. Users reported that the app should provide information that will cater for both mother and child, inclusive of cesarean section birth recovery techniques, a Chat-bot, or numbers of organizations that can offer mental health support for mothers suffering from depression. Additionally, mothers stated that the app should raise awareness about maternal complications, mental health, and techniques that will help mothers regulate and communicate their feelings. A few participants expressed a desire for the RTHA to have information that would encourage women to attend antenatal care appointments during their pregnancy, such as information on body changes, safe exercises, nutrition, and possible health complications to reduce maternal adverse pregnancy outcomes.

Participants reported that the RTHA provides useful information that promotes healthy infant and child nutrition, including tips on breastfeeding and complementary feeding and important developmental milestones. Additionally, participants reported that the RTHA has useful information that will assist both mothers and children, such as information about the required documents to obtain a birth certificate from the South African Department of Home Affairs. Additionally, participants reported that RTHA educates and guides them on the steps to take when babies show different danger signs.

“There is not much information about postnatal care for the mother, it’s mainly about baby, and breastfeeding, and that's all. I think some information for mother should also be included, some women have scissors (caesarean section) and not much information is included, given after on how to care for the wound or what to expect you know if there is a problem with the wound. That’s just an example, so I think including just post-natal care information for the mother would be a great idea”—RTHA-23 (existing user)

 “To give more information on how to deal with depression. Not just mention that there is depression and go speak to the health caretaker. Like make us understand more things like maybe if you can say like I had this suicidal thought where I just wanted to drink pills but luckily, I got over it. For now, I am not sure for how long.`2 So they can at least give you encouraging words or get someone to be there for you even if it’s not them being there for you but words of encouragement that would help”—RTHA-6 (new user)

### Usefulness of the RTHA

Both new users and existing participants perceived the implementation of the RTHA to be useful in providing mothers with more information regarding the vaccines administered to their children and the overall importance and benefits of getting children vaccinated, while at the same time addressing their concerns or questions regarding children’s immunizations. Participants reported that the RTHA could be used to bridge the gap of lack of knowledge in the community, and they perceived that it would help lessen the burden on healthcare professionals, as people would come informed about the vaccine scheduled for a particular appointment. Most participants reported using the RTHA for tracking the vaccination schedule and reported that it would be helpful if the database could be linked with the National Department of Health database to avoid possible complications when the Road to Health Booklet is misplaced. Both existing and new user participants reported that they would appreciate the RTHA sending notifications or reminders for vaccination visits.

 “It is very useful because at the clinic they do not give us enough information. They work in a hurry, they are rushing to finish so they do not really have time even when you ask, they would just say ‘yes’. So, you see when you have this app, you will be able to use the app to get an explanation then you go to the nurses knowing because sometimes they also make mistakes”—RTHA-23 (new user)

 “I mean if that App actually was to be created in such a way that when you put your child’s information automatically its recorded with the department itself you know just like a book you know because sometimes the book gets lost sometimes it gets misplaced you know so if we were able that actually store that information and it works as a book and we are actually able to pick our phones and go to the clinic and say look I’m coming here for immunization 6 months and put my record on the App that would actually great it would enable so many things you know …I’m more convinced that it would definitely work for me”—RTHA-13 (existing user)

## Discussion

Our findings revealed a number of challenges that may have an impact on the willingness to use mobile health tools in specific South African communities. It revealed that although most participants were willing to use the RTHA, there are critical barriers that affected their decision or that of others in the community. The main barriers cited by participants were: (1) access to mobile phones; (2) data costs; (3) app esthetics; (4) exclusive languages used; and, lastly, (5) features and functions. Mothers gave insights on the usefulness and feasibility of the RTHA regarding further innovations to ensure successful implementation of mHealth interventions within the NDoH. In general, participants were receptive to using the RTHA and incorporating it into everyday life to improve prenatal care; however, they were met with some challenges that could make it difficult for consistency and continuity in using the app. Understanding the perspectives of the mothers will assist the NDoH in mitigating some of the barriers present and offering an opportunity for improvement and an update of current mobile phone applications.

Access to smart mobile phones and the costs of mobile data were cited as barriers by participants, although all participants who were enrolled in the study had Android phones, as this was an exclusion criterion. Most participants reported that some of their community members are most likely to not have these devices readily available, which would affect their capability to use the app. According to Statista, in 2021, 44.53 million South Africans had some access to mobile internet using some sort of device (mobile phone, laptop, computer), and that number had risen by 3.26 as of June 2022 (12), revealing that although this is a barrier, access to mobile phones and the internet is steadily increasing in South Africa. Similarly, a study (13) exploring the systematic adoption of mobile health technology named financial and economic barriers as the top three factors affecting the implementation and scaling-up of mobile health tools. Data prices in South Africa are high despite efforts to reduce them, and the unemployment rate in South Africa was cited as being at an all-time high in 2021; and (14) as a result, many people cannot afford to buy data monthly. Although the RTHA is designed to work offline, some participants, especially new users, reported that the app does not work when they do not have mobile data. Innovative technological tools ought to be designed in a way that is user-friendly and considers the economic status of the targeted end-user.

Participants reported that the navigation panel of the RTHA was complicated for some participants who had limited education and that there were little to no graphics to help new users, and this is consistent with a study by (15) that revealed that to improve customer engagement; designers were encouraged to include pictures. Although this study was focused on marketing, it emphasizes the preference for people to see some pictures on the RTHA to improve engagement and provide visual guidance for participants who might find it challenging to read the information presented. One meta-analysis study found that including graphics significantly increased student reading comprehension (16), emphasizing the importance of including esthetically pleasing graphics to improve mothers’ experience. Although this feature could further influence and complicate the mobile phone requirements for downloading as some features and graphics would impose a requirement for a higher Android software (17).

The majority of the participants agreed that the RTHA would need to be offered in additional South African official languages. They reported concerns with diversity and inclusivity for some participants whose languages are not included in the app. Participants noted that this might make other language groups feel discriminated against or excluded. Additionally, participants reported having concerns regarding the level of reading proficiency and comprehension and given the diversity of the community, mothers felt that more languages should be added, as some members of the community may be able to speak and understand more than one language but may be unable to read it. Therefore the concern raised is that of language literacy.

Lastly, the RTHA was reported to have repetitive information and merely acted as an information tool. Moreover, mothers suggested that the app could be used interchangeably with the current Road to Health booklet that mothers present at the clinic during their regular immunization visits. As a result, the NDoH now has the opportunity to scale up the tool and use it to replace the current booklet as the world transitions to digitization (18).

### Recommendations

**Table:** 

BARRIERS	RECOMMENDATIONS
1. MOBILE PHONE AND DATA ACCESS	For the RTHA to be made available offline and at clinics and hospitals in rural areas to allow community mothers without access or opportunity to utilize it.
2. APP USABILITY	Allow the app to offer backup options and fix bugs and errors.
3. APP ESTHETICS	Include more graphics to assist with readability.
4. LANGUAGE	Offer the app in all official South African languages.
5. CONTENT UPDATES	Include personalized information based on child milestones and regularly update information by offering recent news/information updates.
6. FEATURES AND FUNCTIONS	Develop RTHA to be linked to the National Department of Health’s Child Immunization Database.

### Limitations

The findings in this paper cannot necessarily be generalized to all people of similar demographics. However, highlight the experiences of individuals who have had exposure to and used the RTHA. Furthermore, it is important to note that demographics included on mobile phone access do not necessarily represent the population in this region as having a smart phone was an exclusion criteria and participants were not provided with mobile phones and were required to use their own devices, future studies could include these participants and provide mobile phones to further explore perspectives of using the RTHA from a more inclusive sample.

## Conclusion

Our results revealed multiple challenges that could have implications for the willingness to uptake mobile health tools in specific communities in South Africa. The barriers cited have potential to be used to improve future mobile health tools by ensuring that they are developed to be user-friendly and to mitigate community specific barriers. The RTHA is an innovative application that will improve the level of postnatal care in South Africa, and further research is needed to further explore the feasibility and acceptability of utilizing this app during regular clinic visits in South Africa. The results of this study will guide application developers to improve the RTHA, help inform policy around the resources lacking to achieve comprehensive immunization in South Africa and add to existing knowledge about the effectiveness of the Road to Health program.

## Data Availability

The raw data supporting the conclusions of this article will be made available by the authors, upon reasonable request to the corresponding author, JD (dietrichj@phru.co.za).
